# Knowledge Summaries for Comprehensive Breast Cancer Control

**DOI:** 10.1200/JGO.17.00141

**Published:** 2017-11-16

**Authors:** Jo Anne Zujewski, Allison L. Dvaladze, Andre Ilbawi, Benjamin O. Anderson, Silvana Luciani, Lisa Stevens, Julie Torode

**Affiliations:** **Jo Anne Zujewski,** Consultant for Leidos Biomedical Research, Frederick; **Lisa Stevens**, National Cancer Institute, Bethesda, MD, **Benjamin O. Anderson​,** Fred Hutchinson Cancer Research Center; **Allison L. Dvaladze**, University of Washington, Seattle, WA, **Andre M. Ilbawi,** World Health Organization European Observatory on Health Systems and Policies, Brussels, Belgium; **Silvana Luciani**, Pan American Health Organization, Washington, DC; and **Julie Torode**, Union for International Cancer Control, Geneva, Switzerland.

## Abstract

Breast cancer is the most common cancer in women worldwide, affecting > 1.6 million women each year, projected to increase to 2.2 million cases annually by 2025. A disproportionate number of the > 500,000 women who die as a result of breast cancer each year reside in low-resource settings. Breast cancer control is an important component of cancer control planning and women’s health programs, and tools are needed across the care continuum to reduce the cancer burden, especially in low-resource settings. Cancer control planning is complex and multifaceted. Evidence shows that outcomes are improved when prevention, early diagnosis, treatment, and palliation are integrated and synchronously developed within a country/region’s health plan. The Knowledge Summaries for Comprehensive Breast Cancer Control are the product of a multiyear collaboration led by the Union for International Cancer Control, Breast Health Global Initiative, Pan American Health Organization, and Center for Global Health of the US National Cancer Institute. Fourteen knowledge summaries distilled from evidence-based, resource-stratified guidelines, and aligned with WHO guidance on breast cancer control, build a framework for resource prioritization pathways and delivery systems for breast cancer control at four levels of available resources: basic, limited, enhanced, and maximal. Each summary contains relevant content to inform breast cancer policy, clinical care, and advocacy, aiding in the development and implementation of policies and programs. These tools provide a common platform for stakeholders, including policymakers, administrators, clinicians, and advocates to engage in decision making appropriate to their local setting. The goal is to facilitate evidence-based policy actions and urgently advance implementation of an integrated approach to reduce breast cancer mortality and improve quality of life.

## INTRODUCTION

Cancer is one of the leading causes of morbidity and mortality worldwide, with approximately 14 million new cases in 2012.^1^ Cancer is the second leading cause of death globally and was responsible for 8.8 million deaths in 2015.^2^ Globally, nearly one in six deaths is due to cancer. The number of new cases is expected to increase by approximately 70% over the next two decades. The majority of deaths as a result of cancer occur in low- and middle-income countries (LMICs). Breast cancer is the second most common cancer in the world and the most frequent cancer among women, with an estimated 1.67 million new cases diagnosed in 2010 (25% of all cancers). It is the most common cancer in women, both in more- and less-developed regions, with slightly more cancer in less-developed (883,000) than in more-developed (794,000) regions. However, few tools exist to assist policymakers in developing effective breast cancer control programs in LMICs. World Health Assembly resolution WHA70.12, Cancer prevention and control in the context of an integrated approach, calls on governments and the WHO to develop and implement tools to establish and implement comprehensive cancer prevention and control programs, leveraging existing tools for resource-stratified guidance.^3^

Our objective was to produce an integrated set of tools to help guide key policy and breast health program interventions on the basis of existing data in a concise, topic-directed, and evidence-based framework satisfying the WHA70.12 mandate and using accepted frameworks for action articulated by the WHO and other key normative agencies. We developed a toolkit entitled Knowledge Summaries for Breast Cancer (KSBC) that addresses foundational questions and answers in comprehensive breast cancer care across the life course, recognizing that variation exists within and between health systems around the globe. The KSBC toolkit consists of 14 major topics in breast cancer control (Table 1), uses the principles of the Breast Health Global Initiative resource-stratified guidelines^4^ aligned with WHO guidance on breast cancer control,^5,6^^,-^ and is designed to assist policymakers, health care administrators, clinicians, and advocates to engage in decision making appropriate to their local setting in developing a comprehensive breast cancer control. This article briefly summarizes the development process for this toolkit and includes a summary of major contents.

**Table 1:**
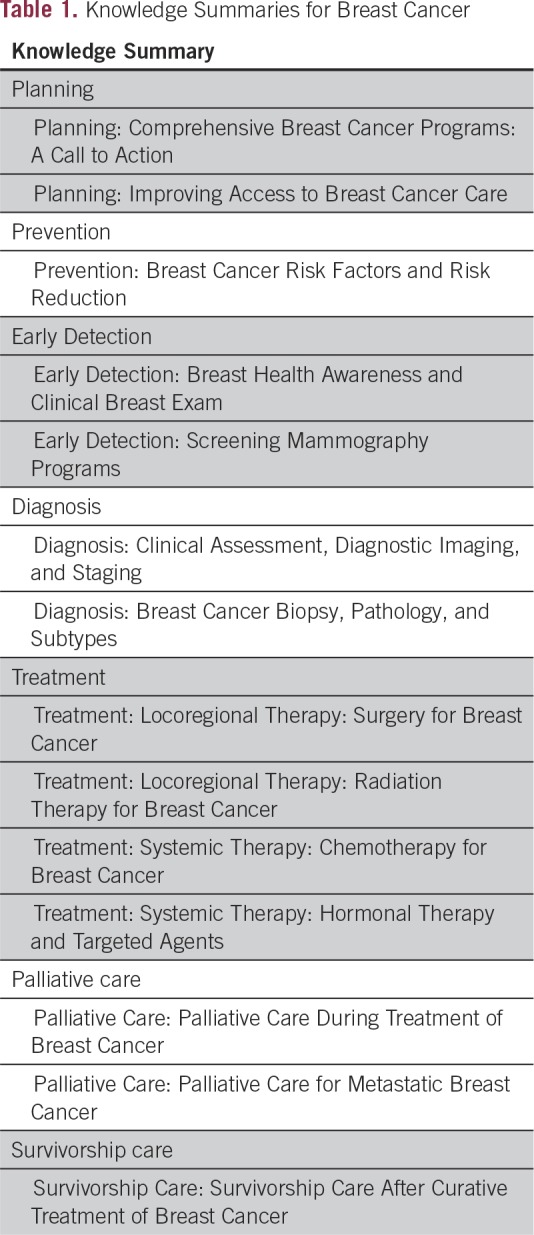
Knowledge Summaries for Breast Cancer

## METHODS

A literature review was conducted using PubMed, PubMed Central, Medline, Cochrane library, and Embase databases. Search terms relevant to each Knowledge Summary (KS) were used in combination, including breast cancer, primary prevention, and/or risk factor reduction for the summary on breast cancer risk factors and risk reduction, focusing on core clinical journals in English. Forward tracking in Web of Science was used to find literature referring to central sources, and bibliographies of recent literature reviews were cross-referenced. To supplement the search, a snowballing strategy was used, searching reference lists to identify relevant studies. Searches were limited to English-language, peer-reviewed publications. More than 1,000 original reports or review articles were identified per summary, and approximately 100 articles were summarized by one member of the development team (A.I.).

Draft documents were reviewed by an external team of volunteers, including experts in medical oncology, surgical oncology, radiation oncology, radiology, public health, health policy, and advocacy. Rewriting, editing, design and approval of the documents were performed by members of the Union for International Cancer Control, the National Cancer Institute Center for Global Health, the Pan American Health Organization, the Breast Health Global Initiative, the Fred Hutchinson Cancer Research Center, and the University of Washington. A preliminary selection of four summaries was released in January 2015 and translated into Spanish for evaluation by health care workers in Lima, Peru. Two were also translated into Russian for evaluation by health care workers, policymakers, and advocates from Central Asia in Tashkent, Uzbekistan in the fall of 2015. This was followed by a focus group of similar professionals from across Africa at the 2015 African Organization for Research and Training in Cancer meeting in Marrakech, Morocco. On the basis of this feedback, the initial KSBC was revised and the series was completed. The full set of revised and redesigned KSBC was launched online on International Women’s Day, March 8, 2017.

## FORMAT

The fourteen KSBC documents span the spectrum of comprehensive breast cancer care modeled after the WHO Cancer Control: Knowledge into Action and the Partnership for Maternal, Newborn and Child Health Knowledge Summaries.^7^ Each KS includes a section directed toward policymakers, called Points for Policymakers, as well as sections directed toward a general audience, including administrators, clinicians, and advocates. Major headings in the policymaker sections are:

- Preplanning
- Planning Step 1: Where are we now?
- Planning step 2: Where do we want to be?
- Planning step 3: How do we get there?

Sections for the general audience are organized with two major headings, What Works and What We Know, and align with the content and structure of the sections for policymakers. By doing so, KSs present an integrated approach to technical knowledge, strategic programs, and related policy. Although comprehensive in scope, each KS is intended to be brief and readable. They focus on providing recommendations for countries with limited resources and tailor recommendations to four different levels of resources. Each KS is written in lay language, with a key summary at the beginning to highlight the more detailed information found in the subsequent pages. The KSBC is available online.^8^

## KNOWLEDGE SUMMARIES

### Planning: A Call to Action

Breast cancer control is most successful when prevention, early diagnosis, treatment, and palliation are integrated and synchronously developed, within the context of a country’s health plan and cancer program. Comprehensive breast cancer care requires an effective health system with trained community health care personnel, including general physicians, nurses, psychologists, social workers, and other specialized professionals. Programs need to use existing resources effectively and integrate community education and early diagnosis programs with accurate diagnostic procedures and timely, accessible, and effective treatments. This summary covers planning for breast cancer programs and introduces the concepts of knowledge summaries and resource-stratified pathways.

### Planning: Improving Access to Care

Improving access to care and reducing disparities in outcomes requires identifying, understanding, and addressing numerous barriers across the cancer care continuum. Barriers can generally be characterized as structural, sociocultural, personal, and financial. Reducing barriers to cancer care services can improve patient outcomes, provided appropriate diagnostic and treatment facilities are available, accessible, and acceptable. This summary discusses how to improve equitable access to breast cancer care by reducing barriers to breast health services.

### Prevention: Breast Cancer Risk Factors and Risk Reduction

Although breast cancer largely cannot be prevented, risks can be reduced. The goal of primary breast cancer prevention is to protect women from developing breast cancer. The goal of secondary breast cancer prevention is to prevent recurrence of breast cancer. Preventive services often receive less attention and funding. However, reducing the incidence of breast cancer through effective community awareness and preventive measures can affect quality of life for women as well as reduce health care expenditures. Experts suggest that if maximal benefit was achieved through prevention and screening programs, 20% to 50% of breast cancers could be avoided.^9^ This summary covers preventive approaches, including prophylactic medications, prophylactic surgery, and lifestyle modifications for breast cancer prevention as well as health professional training and individual risk assessments.

### Early Detection: Breast Health Awareness and Clinical Breast Exam

Early detection of breast cancer begins with the establishment of programs to improve early diagnosis of symptomatic women. Early recognition of symptoms and accurate diagnosis of breast cancer can result in cancers being diagnosed at earlier stages when treatment is more feasible, affordable, and effective. This requires that health systems have trained front-line personnel who are able to recognize the signs and symptoms of breast abnormalities for both benign breast issues as well as cancers, perform an appropriate clinical evaluation, and know the proper referral protocol when diagnostic work-up is warranted. When linked to effective treatment, early diagnosis can lead to better breast cancer outcomes and survival rates. This summary covers the necessary components of breast awareness programs for early diagnosis and the clinical breast evaluation.

### Early Detection: Screening Mammography Programs

Early detection is an important component of a comprehensive breast cancer care strategy. It includes early diagnosis of symptomatic women and may include screening programs offered to asymptomatic women. Screening programs have been shown effective only when the incidence of breast cancer in the target population is high and an accurate diagnosis and effective treatment are universally available and accessible in a timely manner. To date, these conditions exist mainly in high-resource settings. Unless there is universal access to diagnostic and treatment services, even the down-staging of tumors through screening programs of asymptomatic women is not likely to reduce breast cancer mortality. This summary covers mammography screening programs and the necessary health system requirements needed to assure quality mammography programs. However, it also notes that care systems in basic and limited-resource settings should focus efforts on increasing health system capacity for breast cancer diagnosis and treatment of symptomatic women before considering mammography screening.

### Diagnosis: Clinical Assessment, Diagnostic Imaging, and Staging

Breast cancer diagnosis requires an efficient referral process and timely coordination of services. A lack of coordination of care and poor patient access to care can cause delays in definitive diagnosis and initiation of treatment, with the potential to negatively influence outcomes. Breast findings suspicious for cancer require referral for tissue biopsy for definitive diagnosis and imaging studies to determine the stage of cancer. This summary includes a review of services needed for evaluation of a breast complaint, including a medical history and a clinical breast examination, imaging studies, biopsy of suspicious lesions, pathology (histology/cytology) studies, and return visit to review the results of diagnostic studies and to discuss a treatment plan.

### Diagnosis: Breast Cancer Biology, Pathology, and Subtypes

The success of an effective breast health care program is directly related to the availability and quality of breast pathology. Accurate tissue diagnosis is the cornerstone of cancer therapy. There is a critical deficit in pathology services in low-resource settings. As a result, women with breast masses, many of which may not be cancerous, are often subjected to unnecessary surgical procedures. This summary reviews the biology, pathology, and subtypes of breast cancer and emphasizes the need for accurate pathologic diagnosis before initiating treatment.

### Treatment: Locoregional Therapy: Surgery for Breast Cancer

Surgical care is one of the primary treatment modalities for locoregional breast cancer; radiotherapy and systemic therapy are the other primary modalities. Surgical care for breast cancer requires expert surgical training and coordination of care. The type of surgery will depend on the disease stage, tumor characteristics, patient preferences, and resources available for neoadjuvant (preoperative) and adjuvant (postoperative) treatments. This summary discusses surgical approaches for breast cancer treatment.

### Treatment: Locoregional Therapy: Radiation Therapy for Breast Cancer

Radiotherapy is an essential component of the multimodality treatment of breast cancer. In LMICs, where most women present with locally advanced breast cancer, the percentage of women who would benefit from radiotherapy is great, yet the gap between the demand and available supply continues to grow. This summary discusses radiotherapy approaches for the treatment of breast cancer and coordination of treatment plans, including timely referrals that incorporate timely radiotherapy into treatment planning.

### Systemic Therapy: Chemotherapy for Breast Cancer

Chemotherapy plays a central role in the treatment of breast cancer for the majority of patients at all resource levels. Chemotherapy improves survival, reduces recurrence, palliates symptoms of advanced disease, and may improve candidacy for definitive surgery or for breast conservation when used before surgery. The specific recommendations for chemotherapy vary by patient, tumor, cost, and resource availability. This summary reviews chemotherapy options for breast cancer.

### Systemic Therapy: Hormonal Therapy and Targeted Agents

Targeted cancer therapies are drugs or other substances that block the growth of cancer by interfering with specific molecules (molecular targets or receptors) that are involved in the growth, progression, and spread of cancer. Accurate testing for the presence of the estrogen receptor with or without the progesterone receptor is required for the effective use of hormonal therapy, and accurate testing of human epidermal growth factor receptor 2 status is required before consideration of human epidermal growth factor receptor 2–targeted therapy. This summary reviews targeted therapies and notes that a resource stratified approach can help determine appropriate introduction of targeted therapies in a stepwise fashion.

### Palliative Care: Palliative Care During Treatment of Breast Cancer

The WHO defines palliative care as “an approach that improves the quality of life of patients and their families facing the problem associated with life-threatening illness, through the prevention and relief of suffering by means of early identification and impeccable assessment and treatment of pain and other problems, physical, psychosocial and spiritual.”^10^ Palliative care may be referred to as supportive care, symptom management or comfort care. In low-resource settings, the capacity to manage adverse effects and toxicities should be a factor in the selection of treatment options for breast cancer.^9^ This summary reviews the palliative care for the prevention and management of physical as well as psychosocial adverse effects of cancer treatment.

### Palliative Care for Metastatic Breast Cancer

A large percentage of women in low-resource settings who develop breast cancer present with advanced (metastatic) disease. In the majority of these patients, treatment with curative intent is not possible. The survival of patients after a diagnosis of metastatic cancer depends on tumor characteristics and available therapies but ranges from several months to several years; therefore, palliative care represents a substantial contribution to breast cancer programs. The importance of quality and culturally sensitive end-of-life care cannot be understated. This summary addresses palliative care for cancer and its treatment in the advanced disease setting.

### Survivorship Care: Survivorship Care After Curative Treatment of Breast Cancer

Breast cancer survivors are patients who have entered the post-treatment phase after the successful completion of breast cancer therapy with curative intent; longer-term endocrine therapy and/or targeted therapy may continue during survivorship care. Globally, breast cancer survival rates are increasing, creating a new generation of survivors in need of ongoing care and counseling. Evidence suggests that a significant number of people with a cancer diagnosis have unmet informational, psychosocial, and physical needs, which can be effectively addressed through survivorship care interventions. This summary reviews survivorship care services, including treatment of long-term complications, surveillance for cancer recurrence, and counseling on prevention strategies, such as lifestyle modifications.

## USING THE KNOWLEDGE SUMMARIES

KSs are intended to provide the evidence-based foundation for policy and program decision making, ensuring that services are designed and implemented according to resource-appropriate and best practices. Accordingly, KSs can be used by all stakeholders when planning, designing, financing, implementing, or monitoring breast cancer control (Table 2). This includes use by policymakers for national cancer control strategic planning, by governing bodies for the development of diagnosis and management guidelines, by caretakers and clinicians to review evidence-based practices, by public health practitioners to build capacity for breast cancer control within women’s health programs, and by advocates to identify priority cancer control activities. The Breast Cancer Initiative 2.5 campaign,^11^ which includes National Cancer Institute, Union for International Cancer Control, and Breast Health Global Initiative as founding collaborating organizations, continue to use the KS library as educational resources for courses and meetings where education regarding breast health care delivery in LMICs is a specific requirement. The Breast Cancer Initiative 2.5 collaborators will continue to study the utility and efficacy of the KS toolkit, just as was done in Peru, Uzbekistan, and Morocco before the launch on March 8, 2017, findings from which will be used to drive future iterations of the toolkit and may be applied to the development of similar tools for malignancies other than breast cancer.

**Table 2:**
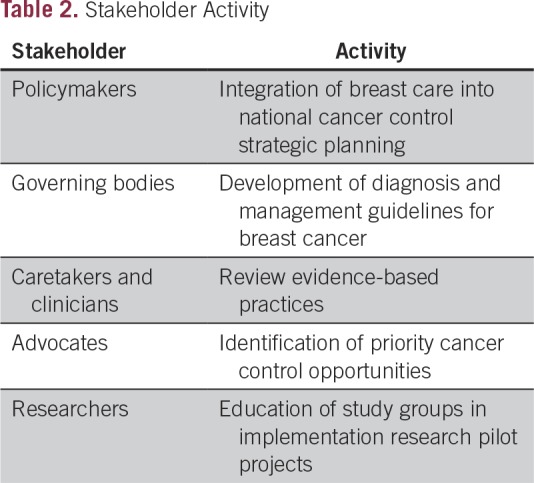
Stakeholder Activity

In conclusion, the KSBC is an evidenced-based toolkit that provides a framework for policymakers, clinicians, and advocates to develop and maintain an integrated breast cancer control program. The KSBC is most helpful when used in conjunction with locally obtained data to develop cancer control plans specific to that country or region of interest. The KSBC is available free of charge.^8^ To ensure that this toolkit remains current, collaborators plan to evaluate the accuracy and impact of the knowledge summaries on an ongoing basis.
